# Associations between the levels of circulating inflammatory adipokines and the risk of type 2 diabetes in Chinese male individuals: A case–control study

**DOI:** 10.1002/jcla.24875

**Published:** 2023-04-01

**Authors:** Xia Sun, Wei‐Wen Qiu, Jing Wu, Shi‐Ling Ding, Rong‐Zhen Wu

**Affiliations:** ^1^ Department of Endocrinology Lishui Hospital of Traditional Chinese Medicine Lishui Zhejiang China; ^2^ Department of Neurology Lishui Hospital of Traditional Chinese Medicine Lishui Zhejiang China; ^3^ Department of Clinical Laboratory Lishui Municipal Central Hospital Lishui Zhejiang China

**Keywords:** case–control study, Chinese male, circulating inflammatory adipokines, type 2 diabetes

## Abstract

**Background:**

Whether the levels of circulating inflammatory adipokines affect the progression of type 2 diabetes (T2D) remains unclear. This study aimed to assess the association between circulating inflammatory adipokine levels and risk of T2D.

**Methods:**

This case–control study involved 130 individuals consisting of 66 healthy controls (Control group) and 64 patients with T2D (T2D group) in Lishui Municipal Central Hospital from January 2017 to June 2017. Multivariate logistic regression analysis was applied to assess the associations between circulating inflammatory adipokine levels and the risk of T2D.

**Results:**

There were significant differences in the levels of adiponectin (*p* = 0.013) and visfatin (*p* < 0.001) between the T2D and Control groups. In contrast, no significant differences in leptin (*p* = 0.113), TNF‐α (*p* = 0.632), and IL‐6 (*p* = 0.156) levels were found between the groups. Multivariate logistic regression indicated that elevated visfatin level was associated with an increased risk of T2D (OR: 3.543; 95% CI: 1.771–7.088; *p* < 0.001), while adiponectin (OR: 1.946; 95% CI: 0.925–4.094; *p* = 0.079), leptin (OR: 3.723; 95% CI: 0.788–17.583; *p* = 0.097), TNF‐α (OR: 1.081; 95% CI: 0.911–1.281; *p* = 0.373), and IL‐6 (OR: 0.878; 95% CI: 0.657–1.173; *p* = 0.379) were not associated with the risk of T2D.

**Conclusions:**

This study found elevated visfatin levels are associated with an increased risk of T2D, while adiponectin, leptin, TNF‐α, and IL‐6 are not. These findings should be further verified by a large‐scale prospective study.

## Graphical abstract

The levels of circulating inflammatory adipokines associated with the risk of T2D were first explored. Elevated visfatin levels were associated with an increased risk of T2D. The causality relationship between circulating inflammatory adipokines and T2D was not obtained owing to cross‐sectional design. Background treatments for T2D were not available, which may have affected the levels of circulating inflammatory adipokines

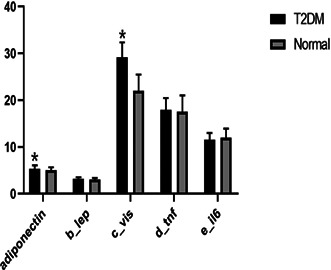

## 1 INTRODUCTION

Currently, there are approximately 463 million patients suffering from diabetes worldwide, with nearly 116.4 million in China, which is expected to increase to 140.5 million in 2030.^1^, ^2^ This global epidemic may be explained by current sedentary lifestyles and unhealthy diets, causes substantial economic burden, and is threatening public health.^3^, ^4^ Type 2 diabetes (T2D), which accounts for more than 90% of diabetic cases, is characterized by a pancreas that cannot produce sufficient insulin.^5^ Studies have found that the progression of T2D is dependent on the interactions between genetic, environmental, lifestyle, and other risk factors.^6^, ^7^, ^8^ These studies have focused on modifiable health behavior factors, while the role of circulating inflammatory adipokine levels in the progression of T2D remains unclear.

Several studies have already explored the potential role of circulating inflammatory adipokines in the development of T2D.^9^, ^10^, ^11^, ^12^, ^13^ Nielsen et al.^9^ examined 30,045 individuals from the Copenhagen General Population Study and found that low plasma adiponectin levels are associated with an increased risk of T2D. Kazmi et al.^10^ performed an observational study and found that low leptin levels are associated with T2D independently from changes in body mass index. Kang et al.^11^ found that visfatin synthesis can be activated in adipose tissue under diabetic conditions through the induction of NF‐κB activation and leads to the production of pro‐inflammatory cytokines and systemic inflammation. Katsuki et al.^12^ found that serum TNF‐α levels play an important role in obesity‐related insulin resistance. Wang et al.^13^ performed a meta‐analysis and found that elevated IL‐6 levels were associated with an increased risk of T2D. However, it remains unclear whether the levels of circulating inflammatory adipokines are associated with the risk of T2D. Therefore, we performed this study to explore the associations between circulating inflammatory adipokine levels and the risk of T2D.

## 2 METHODS

### 2.1 Patients

Sixty‐six healthy (Control group) and 64 T2D (T2D group) male individuals were recruited at the Lishui Municipal Central Hospital from January 2017 to June 2017. Individuals at T2D and control groups were matched by the body mass index (BMI). A minimal sample size of 60 for each group was calculated based on the 1:1 case–control design (α = 0.05, 1–β = 0.8, and odds ratio, OR > 3.0).^14^ T2D was diagnosed according to the criteria of ADA,^15^ and male individuals aged 18.0 years or older were eligible in our study. Individuals presenting the following characteristics were excluded: (1) acute diabetic complications; (2) pulmonary or cardiac lesions; (3) liver or kidney disorders; and (4) overweight or obesity caused by other diseases. The Declaration of Helsinki principles was applied to guide this study, and the study was approved by the Institutional Review Board of Lishui Hospital of Traditional Chinese Medicine (No: 2021‐LW‐013). All participants signed the informed consents after being explained the purpose of this study (Appendix S1).

### 2.2 Measurement of circulating inflammatory adipokine levels

Elbow venous blood was taken from the patients after 12 h of overnight fasting. Fasting adiponectin levels were measured by an enzyme‐linked immunosorbent assay (ELISA) kit (catalogue number ab222508). The ELISA plate reader (Tecan Sunrise Reader, 96‐well Microplate Reader) was applied to read the absorbance at 450 nm wavelength. Commercial radioimmunoassay kits (catalog number ab179884)were used to measure plasma leptin levels, with a standard curve range of 0–50 ng/mL. A visfatin enzyme immunoassay kit (catalogue number ab264623) was used to measure visfatin, with a standard curve range of 0.1–1000 ng/mL. Serum levels of TNF‐⍺ and IL‐6 were assessed using the ELISA kit (Jiangsu Meimian Industrial Co., Ltd, Jiangsu, China). All measurements were conducted in Shanghai Enzyme Linked Biotechnology Co., LTD.

### 2.3 Baseline anthropometric, clinical, and biochemical variables

Trained staff measured the anthropometric variables, and weight or height was assessed with light clothing and no shoes using calibrated scales and a wall‐mounted stadiometer. BMI was calculated using weight and height with the units kg/m^2^. Overweight was defined as BMI ≥25 kg/m^2^. An anthropometric tape was used to measure waist circumference, hip circumference, and waist–hip ratio after normal exhalation. Body fat and visceral fat were assessed using InBody 720 Body composition analyzer (Shanghai Bass Medical Instrument Trading Co., LTD). Routine laboratory analyses were performed after 12 h of overnight fasting: triglyceride (TG), total cholesterol (TC), high density lipoprotein‐cholesterol (HDL‐C), low density lipoprotein‐cholesterol (LDL‐C), and fasting blood glucose (FBG) were assessed using AU5800 chemistry analyzer (Beckman Coulter, CA, USA) with the appropriate kits. Hemoglobin A1c (HbA1c) was assessed using high‐performance liquid chromatography (ARKRAY, Kyoto, Japan). Insulin was measured using chemiluminescent immunoassay (Beckman Coulter, CA, USA). The Serum TSH, FT3, and FT4 levels were examined by chemiluminescence immunoassay (ADVIA Centaur chemiluminescence immunoanalyzer, Siemens Healthcare Diagnostics Inc., Erlangen, Germany) with a mating reagent was applied to measure serum‐free triiodothyronine (FT3), free thyroxine (FT4), and thyroid stimulating hormone (TSH). The gamma‐glutamyl transpeptidase (GGT) and uric acid were assessed using the standard enzymatic methods (Beckman Coulter, CA, USA). A validated semi‐automatic oscillometer (Omron HEM‐70CP; Hoofddorp, The Netherlands) was used to measure the blood pressure of seated individuals by trained staff. Mean arterial pressure (MAP) was calculated as MAP = 1/3*systolic blood pressure + 2/3*diastolic blood pressure.^16^ The homeostasis model assessment of insulin resistance (HOMA‐IR) index was calculated using a standard equation based on plasma insulin and plasma glucose levels.^17^

### 2.4 Statistical analysis

All variables were classified as continuous or categorical. Data from continuous variables were displayed as mean ± standard deviation or median (quartile) based on data distribution. Data from categorical variables were displayed as frequency and proportion. Then, Student's *t*‐test and Kruskal–Wallis tests were applied to compare the differences between patients with T2D and healthy controls for continuous data normally or abnormally distributed. The chi‐square test was used to compare the differences between groups for categorical data. Univariate logistic regression analysis was applied to identify potential risk factors for T2D and determine the factors to be subjected to the multivariate logistic regression analysis. All tests were double‐tailed, and statistical significance was set at *p* < 0.05. IBM SPSS Statistics for Windows, version 19.0 (SPSS 19.0) was used to perform statistical analysis.

### 2.5 Patient and public involvement

This project did not include patient or public involvement in developing the research questions, design, conduct, and choice of outcome measures or recruitment.

## 3 RESULTS

### 3.1 Baseline characteristics

The baseline characteristics of the participants are shown in Table 1. There were significant differences between the T2D and Control groups for age (*p* < 0.001), TSH (*p* = 0.019), FT3 (*p* < 0.001), FBG (*p* < 0.001), HOMA‐IR (*p* < 0.001), TC (*p* = 0.004), HDL‐C (*p* < 0.001), LDL‐C (*p* = 0.013), homocysteine (*p* < 0.001), uric acid (*p* = 0.005), MAP (*p* = 0.017), and HbA1c (*p* < 0.001) levels, waist circumference (*p* = 0.025), waist‐hip ratio (*p* < 0.001), and visceral fat (*p* = 0.010). However, there were no significant differences between groups for overweight (*p* = 0.863), FT4 (*p* = 0.186), fasting insulin (*p* = 0.153), GGT (*p* = 0.966), and TG (*p* = 0.505) levels, hip circumference (*p* = 0.778), and body fat (*p* = 0.557).

**TABLE 1:** The baseline characteristics of included patients.

Variable	Normal	T2D	*p* value
Age (years)	43.00 (39.00, 50.00)	50.00 (45.00, 54.50)	<0.001
Overweight	34 (51.52)	32 (50.00)	0.863
TSH (mU/L)	1.52 (1.31, 2.19)	1.32 (0.94, 2.00)	0.019
FT3 (pg/mL)	3.10 (2.95, 3.31)	2.59 (2.30, 2.87)	<0.001
FT4 (ng/dL)	1.06 (0.98, 1.12)	1.02 (0.92, 1.12)	0.186
FBG (mmol/L)	5.34 (5.06, 5.68)	8.62 (6.97, 12.55)	<0.001
Fasting insulin (μU/mL)	7.15 (4.90, 11.60)	5.60 (4.50, 8.95)	0.153
HOMA‐IR	1.70 (1.20, 2.73)	2.94 (1.56, 4.35)	<0.001
GGT (U/L)	30.50 (22.00, 72.00)	34.00 (20.00, 60.00)	0.966
TG (mmol/L)	1.61 (1.08, 2.51)	1.44 (1.02, 2.22)	0.505
TC (mmol/L)	5.05 (4.50, 5.59)	4.47 (3.92, 5.07)	0.004
HDL‐C (mmol/L)	1.19 (1.07, 1.36)	1.01 (0.84, 1.17)	<0.001
LDL‐C (mmol/L)	2.95 (2.51, 3.45)	2.63 (2.16, 3.14)	0.013
HCY (μmol/L)	10.55 (9.10, 12.60)	8.80 (8.20, 9.80)	<0.001
Uric acid (μmol/L)	348.00 (317.00, 389.00)	307.00 (274.00, 366.00)	0.005
MAP (mmHg)	92.00 (85.00, 97.00)	94.50 (89.00, 105.50)	0.017
HbA1c (%)	5.40 (5.30, 5.70)	10.10 (7.80, 11.30)	<0.001
Waist circumference (cm)	86.50 (80.00, 91.00)	90.00 (83.00, 94.00)	0.025
Hip circumference (cm)	95.50 (93.00, 100.00)	96.00 (92.00, 99.00)	0.778
Waist‐hip ratio	0.89 ± 0.05	0.93 ± 0.05	<0.001
Body fat (%)	23.23 ± 6.59	23.88 ± 5.85	0.557
Visceral fat (cm^2^)	98.50 ± 26.10	110.81 ± 27.65	0.010

Abbreviations: FBG, fasting blood glucose; FT3, free triiodothyronine; FT4, free thyroxine; GGT, gamma‐glutamyl transpeptidase; HCY, hemocysteine; HDL‐C, high density lipoprotein‐cholesterol; HOMA‐IR, homeostasis model assessment of insulin resistance; LDL‐C, low density lipoprotein‐cholesterol; MAP, mean arterial pressure; TC, total cholesterol; TG, triglyceride; TSH, thyroid stimulating hormone.

### 3.2 Circulating inflammatory adipokines

**FIGURE 1:**
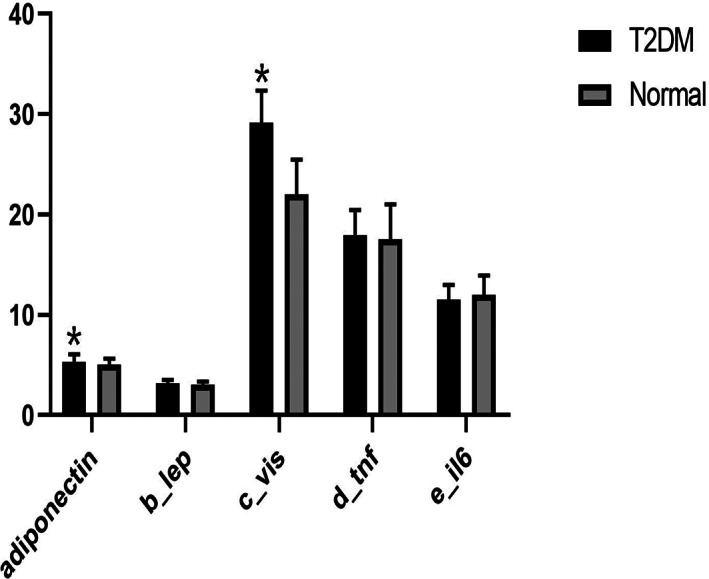
The circulating inflammatory adipokine levels in patients with T2D and healthy controls. b_lep: leptin; c_vis: visfatin; d_tnf: TNF‐α; e_il6: IL‐6. *p < 0.05.

Circulating inflammatory adipokine levels in the T2D and Control groups are shown in Figure 1 and Table 2. Adiponectin and visfatin levels in the T2D group were significantly higher than those in the Control group (adiponectin: 5.34 ± 0.73 vs 5.04 ± 0.63, *p* = 0.013; Visfatin: 29.16 ± 3.21 vs 22.01 ± 3.48, *p* < 0.001). In contrast, no significant differences between the groups were found for leptin (*p* = 0.113), TNF‐α (*p* = 0.632), or IL‐6 (*p* = 0.156) levels.

**TABLE 2:** The circulating inflammatory adipokines levels in T2D and control groups.

Variable	Normal	T2D	*p* value
Adiponectin (μg/mL)	5.04 ± 0.63	5.34 ± 0.73	0.013
Leptin (ng/mL)	3.05 ± 0.32	3.15 ± 0.36	0.113
Visfatin (ng/mL)	22.01 ± 3.48	29.16 ± 3.21	<0.001
TNF‐α (fmol/mL)	17.54 ± 3.47	17.92 ± 2.53	0.632
IL‐6 (pg/mL)	11.99 ± 1.92	11.53 ± 1.45	0.156

### 3.3 Risk factors for T2D

The results of multivariate logistic regression analysis are shown in Table 3. We found that high visfatin levels were associated with an increased risk of T2D (OR: 3.543; 95% CI: 1.771–7.088; *p* < 0.001). However, the levels of adiponectin (OR: 1.946; 95% CI: 0.925–4.094; *p* = 0.079), leptin (OR: 3.723; 95% CI: 0.788–17.583; *p* = 0.097), TNF‐α (OR: 1.081; 95% CI: 0.911–1.281; *p* = 0.373), and IL‐6 (OR: 0.878; 95% CI: 0.657–1.173; *p* = 0.379) were not associated with the risk of T2D.

**TABLE 3:** Multivariate logistic regression for the risk of T2D.

Variable	β	Se	Statistic	*p* value	OR	ORL	ORU
Adiponectin	0.666	0.380	3.079	0.079	1.946	0.925	4.094
Leptin	1.315	0.792	2.755	0.097	3.723	0.788	17.583
Visfatin	1.265	0.354	12.789	<0.001	3.543	1.771	7.088
TNF‐α	0.078	0.087	0.795	0.373	1.081	0.911	1.281
IL‐6	−0.130	0.148	0.773	0.379	0.878	0.657	1.173

## 4 DISCUSSION

The prevalence of diabetes is rising at an alarming rate. Currently, T2D accounts for more than 90% of diabetic cases. Genetic, environmental, lifestyle, and other risk factors are known to play a role in the development of T2D; however, the role of circulating inflammatory adipokine levels on the progression of T2D remains unclear. Here, we evaluated the associations between circulating inflammatory adipokine levels and the risk of T2D. A total of 130 Chinese male individuals, including 66 healthy controls and 64 patients, with T2D were involved, and a wide range of characteristics were examined. The major findings of the current study are that adiponectin and visfatin levels in the T2D group were higher than those in the healthy control group. Moreover, elevated visfatin levels were associated with an increased risk of T2D, while adiponectin, leptin, TNF‐α, and IL‐6 levels were not associated with the risk of T2D.

Our study found that elevated visfatin levels are significantly related to a high risk of T2D. Several studies have already illustrated the potential role of visfatin in T2D.^18^, ^19^, ^20^ Esteghamati et al.^18^ performed a case–control study involving 76 newly diagnosed patients with T2D and 76 healthy control subjects and found that serum visfatin levels in the T2D group were higher than those in the control group. Moreover, serum visfatin levels were associated with an increased risk of T2D after adjusting for obesity parameters. Mabrouk et al.^19^ found that the concentration of serum visfatin is positively correlated with insulin levels and HOMA‐IR index. Kaminska et al.^20^ found that serum visfatin levels were significantly related to TG, HbA1c, and C‐peptide levels as well as waist–hip ratio. Li et al.^21^ indicated plasma visfatin levels are significantly associated with HbA1c and 2‐h oral glucose tolerance test. Beta‐cell deterioration in patients with newly diagnosed T2D may increase serum visfatin levels.^22^ Visfatin is involved in the inflammatory process, and glucocorticords can induce visfatin release from adipocytes.^23^ Interestingly, our study did not find significant association between IL‐6 level and T2D risk. The reason for this may be that IL‐6 can inhibit the transcription of visfatin, and serum IL‐6 levels are negatively correlated with fasting insulin levels and the HOMA‐IR index.^24^

The current study found that serum adiponectin levels in the T2D group were higher than those in the control group, while after adjusting for potential confounders, adiponectin was not associated with the risk of T2D. These results are inconsistent with those of previous studies.^9^, ^25^ Nielsen et al observed 756,219 individuals and found a 1 μg/mL low plasma adiponectin was associated with an increased risk of T2D.^9^ Yasuda et al identified 107 obese children and found plasma adiponectin levels in T2D group are significantly lower than those of the metabolic syndrome and healthy groups. These studies indicated that adiponectin has anti‐diabetic and anti‐arteriosclerotic effects, and that lower adiponectin levels are associated with elevated insulin resistance.^26^ A study conducted by Wang et al identified 571 T2D cases and 571 healthy controls and found highest versus lowest tertile of adiponectin levels was associated with a reduced risk of T2D.^27^ However, considering the individuals' ages are differing between our study and previous studies.^9^, ^25^ Similarly, we did not observe a significant association between leptin levels and the risk of T2D, which could be explained by the age differences in the T2D and control groups. This may have biased the relationship between adiponectin or leptin and the risk of T2D.

A study performed by Katsuki et al.^12^ showed that patients with T2D and obesity had lower TNF‐α levels after treatment, while the levels of TNF‐α were unchanged in patients without obesity. Another study found that TNF‐α can inhibit insulin action and play an important role in obesity‐derived insulin resistance.^28^ Moreover, the transport of glucose in an autocrine or paracrine fashion may be inhibited by the overexpression of TNF‐α in adipose tissue. However, our study did not find a significant association between TNF‐α levels and the risk of T2D. Nevertheless, the variation in TNF‐α levels between the participants was small; therefore, further verifications are needed.

Our study have several strengths: (1) individuals at T2D and control groups were matched by BMI, which could eliminate the potential impacts of obesity on circulating inflammatory adipokines; and (2) analyzed using both univariate and multivariate analyses, and the potential confounders were adjusted. Despite the above strengths, the limitations of this study should be acknowledged: (1) this was a case–control study and, therefore, causality could not be inferred; (2) we did not address the treatments that the patients with T2D were undergoing, which may have affected the levels of circulating inflammatory adipokines; (3) stratified analysis was not performed due to the small number of included participants; and (4) the analysis was based on continuous variables, and cutoff values for circulating inflammatory adipokines were not calculated.

## 5 CONCLUSION

In conclusion, this study found that adiponectin and visfatin levels in the T2D group were higher than those in the control group. After adjusting for potential confounders, visfatin level may be associated with an increased risk of T2D. The results of this study should be further verified by a large‐scale prospective study.

## AUTHOR CONTRIBUTIONS

Xia Sun, Wei‐Wen Qiu, Jing Wu, Shi‐Ling Ding, and Rong‐Zhen Wu contributed equally to this work. All authors reviewed and revised the manuscript and provided approval of the final version submitted for publication.

## FUNDING INFORMATION

The present study was supported by the Health Commission of Zhejiang Province (2020KY1089).

## CONFLICT OF INTEREST STATEMENT

The authors declare that they have no competing interests.

## PATIENT CONSENT STATEMENT

All persons gave their informed consent prior to their inclusion in the study.

## Supporting information


Appendix S1.

## Data Availability

The data presented in this study are available on request from the corresponding author. The data are not publicly available due to patient privacy.
